# Downregulation of SENP1 impairs nuclear condensation of MEF2C and deteriorates ischemic cardiomyopathy

**DOI:** 10.1002/ctm2.70318

**Published:** 2025-05-07

**Authors:** Ying Xie, Qiaoyuan Li, Xiyun Bian, Yan Yin, Zhuo Liang, Xu Liu, Tao Zhang, Xiaozhi Liu, Xin Quan, Yunlong Wang

**Affiliations:** ^1^ Shandong Provincial Key Laboratory of Precision Oncology Shandong Cancer Hospital and Institute Shandong First Medical University and Shandong Academy of Medical Sciences Jinan Shandong China; ^2^ Department of Cardiology Beijing Anzhen Hospital, Capital Medical University Beijing China; ^3^ Tianjin Key Laboratory of Epigenetics for Organ Development in Preterm Infants The Tianjin Fifth Central Hospital, Binhai Tianjin China; ^4^ Ultrasound Imaging Center Fuwai Hospital, National Center for Cardiovascular Disease Chinese Academy of Medical Sciences and Peking Union Medical College Beijing China

**Keywords:** DeSUMOylation, ischemic cardiomyopathy, MEF2C, protein phase separation, SENP1

## Abstract

Ischemic cardiomyopathy (ICM) is characterised by the insufficient capacity of the heart to effectively pump blood, which ultimately contributes to heart failure (HF). In this study, the down regulation of SENP1 is identified in the cardiomyocyte of ICM mouse models and in patients. The depletion of SENP1 exacerbates hypoxia‐induced apoptosis of cardiomyocytes in vitro and deteriorated cardiomyocyte injury of ICM mice in vivo. Mechanistically, SENP1 deSUMOylates the SUMO2‐mediated modification of MEF2C at lysine 401 for stabilising protein stability. Moreover, the interaction with SENP1 controls the nuclear condensation of MEF2C to promote the expression of genes critical for cardiomyocyte function. When rescuing SENP1 expression using adeno‐associated virus serotype 9, the attenuation of cardiomyocyte injury is discerned in the mouse model of ICM. Therefore, these finding elicits a previously unrecognised role and mechanism of SENP1 in safeguarding cardiomyocyte in ICM progression while establishing a basis for the development of SENP1 as a potential marker for ICM diagnosis and treatment.

**Key points:**

- SNEP1 is downregulated in the cardiomyocyte of ICM mouse models and in patients.
- SENP1 deSUMOylates the SUMO2‐mediated modification of MEF2C at lysine 401 for protein stability.
- The interaction with SENP1 controls the nuclear condensation of MEF2C to promote cardiomyocyte function.
- Cardiac rescue of SENP1 alleviates ischemic heart injury in ICM mouse models by AAV9.

## Graphical abstract

1. SNEP1 is downregulated in the cardiomyocyte of ICM mouse models and in patients.
2. SENP1 deSUMOylates the SUMO2‐mediated modification of MEF2C at lysine 401 for protein stability.
3. The interaction with SENP1 controls the nuclear condensation of MEF2C to promote cardiomyocyte function.
4. Cardiac rescue of SENP1 alleviates ischemic heart injury in ICM mouse models by AAV9.

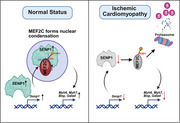

## 1 INTRODUCTION

Ischemia plays a crucial role in heart failure (HF) syndromes, a significant cause of mortality worldwide, and this burden increases with the aging of the population.^1^, ^2^ The worldwide incidence of ischemic cardiomyopathy is increasing, currently standing at 1655 cases per 100 000 individuals, with projections suggesting it will surpass 1845 by 2030.^3^ Despite extensive research on the pathophysiology of ICM over the past decades, the molecular mechanisms underlying it remain unclear. Discovering an effective way to protect cardiomyocytes during ischemic injury is a key objective that could help prevent the progression to HF and lead to the development of new treatment strategies, significantly improving survival rates.

SUMOylation is an essential posttranslational modification that controls the accumulation and functions of proteins and has been shown to be intimately involved in various signalling pathways that influence the fate of cardiomyocytes.^4^, ^5^ Small ubiquitin‐like modifiers (SUMOs) can be conjugated to target proteins in either monomeric or polymeric forms.^6^ SUMO molecules can be easily removed by the six members of the SUMO‐specific protease (SENP) family, which includes SENP 1–3 and SENP 5–7.^7^ They contribute to the functional plasticity and fate of proteins.^8^ SENP1, among SENPs, plays a significant role in removing SUMO1 and SUMO2/3 modifications from numerous target proteins, thereby participating in a wide array of cellular processes.^9^ Variations in the expression and activities of SUMOylation cascade players and defects in certain SUMO substrates have been reported to be related to the pathophysiology of cardiovascular diseases.^4^ For example, Wang et al. demonstrated that Sumo1‐deficient mice suffer from atrial and ventricular septal defects, which contribute to their premature death.^10^ Another study demonstrated that myocardial ischemia‐reperfusion injury increases SENP1 levels, which plays a cardioprotective role by activating the hypoxia‐inducible factor 1α (HIF1α) pathway.^11^ However, knowledge about how the SUMOylation system is regulated and the possible target proteins implicated in ischemic cardiomyopathy (ICM) is still insufficient. This highlights the pressing need to deepen our understanding of SUMOylation in ICM and to create effective therapeutic approaches.

Transcription factors such as MEF2C, GATA4, TBX5, and MYOCARDIN have been identified as playing crucial roles in the differentiation of cardiomyocytes and in various cardiovascular pathological processes.^12^, ^13^, ^14^ For instance, the overexpression of MEF2C in resident cardiac fibroblasts has been shown to promote myocardial regeneration and potentially facilitate the repair of chronic myocardial infarction.^15^ Notably, several of these proteins have been indicated to be influenced by SUMOylation.^12^, ^14^, ^16^ The transcriptional function of Myocardin (MYOCD) is reliant on SUMO1/PIAS1‐mediated SUMO modification.^14^  While significant advancements have been achieved in the development of molecules capable of inhibiting components within the SUMOylation cascade,^17^ none of these have yet been clinically employed for the treatment of cardiovascular diseases, particularly heart failure and myocardial infarction.^18^ Given the association of SUMOylation with the regulation of cardiac function, it is imperative to identify additional instances of SUMO conjugation involving transcription factors and to deepen our comprehension of the interplay between transcription factor dysfunction and altered SUMOylation. Furthermore, there is an ongoing challenge in translating current knowledge into novel diagnostics and therapies.

In the current study, we have identified SENP1 as a significantly downregulated gene in cardiomyocytes exposed to hypoxic conditions using RNA‐seq analysis. Through a series of in vivo and in vitro experiments, we have unveiled the protective role of SENP1 in mitigating ischemic injury in cardiomyocytes. Our study delved into the molecular mechanisms by which SENP1 regulates the stability and LLPS formation of MEF2C. Additionally, we assessed the therapeutic potential of adeno‐associated virus 9 (AAV9)‐mediated *Senp1* gene delivery in improving cardiac pathology and reversing postischemic heart failure in a preclinical model of heart failure. Overall, our findings underscore the promise of SENP1 overexpression as a potential therapeutic strategy for cardiovascular diseases.

## 2 METHODS AND MATERIALS

### 2.1 Animal ethical approval

Animal study was approved by the Ethic Committees of the Tianjin Fifth Central Hospital (No. TJWZX2020011) and conformed to the Guide for the Care and Use of Laboratory Animals published by the US National Institutes of Health (Publication No. 85‐23, revised 1996). C57BL/6 mice were obtained from SPF Beijing Biotechnology. *Myh6‐MerCreMer* mice were purchased from Cyagen Biosciences, and the *Senp1* ^fl/fl^ mice were provided by Zhiqiang Liu, Tianjin Medical University.^19^ *SENP1^fl/fl^; Myh6‐MerCreMer* (*Senp1*‐TKO) were produced by mating *Senp1* ^fl/fl^ mice to *Myh6‐MerCreMer* mice. Mice aged 6–8 weeks and matched for sex were used in this study. The primers for *Senp1* ^fl/fl^ were: loxP forward, 5′‐AGAGTGAGACCCTGTCTCAACCCAAGC‐3′ and loxP reverse 1, 5′‐ CACACAACTAAGTTAACTGCTGGAAACCAGAGC‐3′, loxP reverse 2: 5′‐GTGTGGTATGTGCATGTGTTGACGCACACG‐3′ with the expected positions in 300 and 260 bps for *Senp1* ^fl/fl^ mice and wild‐type mice.

### 2.2 Human material

Human heart tissues were excised from the left ventricular of patients receiving heart transplant surgeries due to ischemic cardiac disease‐induced heart failure in the Department of Cardiac Surgery, the Tianjin Fifth Central Hospital, and ventricular samples from patients who received atria valve replacement surgery was used as the control. Information of patients enrolled in this study was presented in the Table S1. The study was approved by the Medical Ethics Committee of the Tianjin Fifth Central Hospital (No. TJWZXLL2022017). Informed consents were obtained from all the transplant patients and from the family members of donors before tissue collection. All studies were performed in accordance with the Declaration of Helsinki.

### 2.3 Mice model for ischemic cardiomyopathy (ICM)

The ICM model was performed on 8‐week‐old male C57BL/6 mice by permanent (14‐day post‐ICM) ligation of the left anterior descending coronary artery as described previously.^20^ In short, mice were anaesthetised using 1% isoflurane, along with a single intraperitoneal injection of 80 mg/kg ketamine and 7 mg/kg xylazine. The mice were subsequently intubated using a 16‐gauge intravenous catheter for mechanical ventilation and connected to a rodent ventilator to ensure a stable respiratory rate. Following the exposure of the heart, the left anterior descending coronary artery was ligated permanently using 7/0 nylon suture. Sham‐operated mice underwent the same surgical procedure without artery ligation. Afterward, the muscle tissue and skin were sutured, and the mice were placed on a heating pad until they recovered from anaesthesia. Before the sacrifice, blood samples were collected, myocardial tissue samples were obtained, TTC staining was conducted to assess the infarct area, masson's staining on myocardial cells was performed to assess the degree of myocardial fibrosis, the weight of the myocardium was measured and the volume of myocardial cells was analysed using ImageJ software.

### 2.4 Fluorescence recovery after photobleaching (FRAP)

H9c2 cells transfected with corresponding plasmids and the purified protein of interest were utilised in FRAP experiments conducted on an Olympus FV1000 IX81‐SIM Confocal Microscope (Olympus, Tokyo, Japan). Photobleaching was carried out using the tornado mode with a 488 nm laser set at 45% power for GFP. The recovery of fluorescence was observed using the 488 nm laser in free‐run mode at intervals of 1–4.2 s. Additionally, fluorescence from an unbleached area within the same field was monitored as a control. The signal was quantified as a ratio relative to the fluorescence intensity prior to photobleaching.

### 2.5 AAV9‐SENP1 cloning, virus packaging and delivery

We produced AAV9‐SENP1 and AAV9‐Mock constructs utilising the cardiac‐specific TNNT2 promoter to drive their expression specifically in the heart. The cDNA fragments encoding SENP1 were individually inserted into AAV9 plasmids containing Inverted Terminal Repeats (ITRs) and the chicken cardiac TNT promoter obtained from the Penn Vector Core. The AAV9 constructs were then packaged in HEK293T cells. After three days, the cells were harvested, lysed, and the AAV particles were purified and concentrated using gradient centrifugation. The quantification of AAV viral particles was assessed by measuring the viral genome count using RT‐qPCR, resulting in titres ranging from 1 × 10^13^ to 4 × 10^13^ viral genome (vg) particles per mL. The Senp1 virus was administered to the mouse heart through tail vein injection, with a viral titre of 1×10^12^ vg/mL and a volume of 100 µL per mouse. The effectiveness of the viral infection was assessed after a duration of 3 weeks and ICM model was established, and 6 mice per group was randomly selected to confirm the Senp1 expression.

### 2.6 Co‐immunoprecipitation (Co‐IP)

Cells were gathered and then lysed with NP‐40 lysis buffer, which included complete protease inhibitors, while kept on ice for 30 min. The cell lysate was subsequently centrifuged at 12 000 × *g* for 20 min at a temperature of 4°C. To co‐immunoprecipitate exogenously expressed proteins, the supernatant was incubated with anti‐FLAG M2 Affinity Gel (Sigma‐Aldrich, A2220) overnight at 4°C. The next day, the pellet was washed four times using NP‐40 lysis buffer buffer before undergoing western blotting analysis.

### 2.7 Statistical analysis

For comparisons between two groups, analyses were conducted using the unpaired Student's *t*‐test (for normally distributed data) or the Mann–Whitney test (for non‐normally distributed data). For comparisons involving multiple groups, one‐way analysis of variance (ANOVA) was applied, followed by Tukey's post hoc test, or two‐way ANOVA with Sidak's post hoc test. When comparing two conditions between groups, two‐way ANOVA with the appropriate post hoc correction was used. The correlation between gene expressions was determined by the Pearson correlation test, and survival analysis was performed using GraphPad Prism 5.0. All the experiments were biologically repeated in at least 3 independent samples, and technically repeated at least 3 times. Statistical significance was considered for *p* values less than  .05.

## 3 RESULTS

### 3.1 SENP1 was downregulated in ICM mice

**FIGURE 1:**
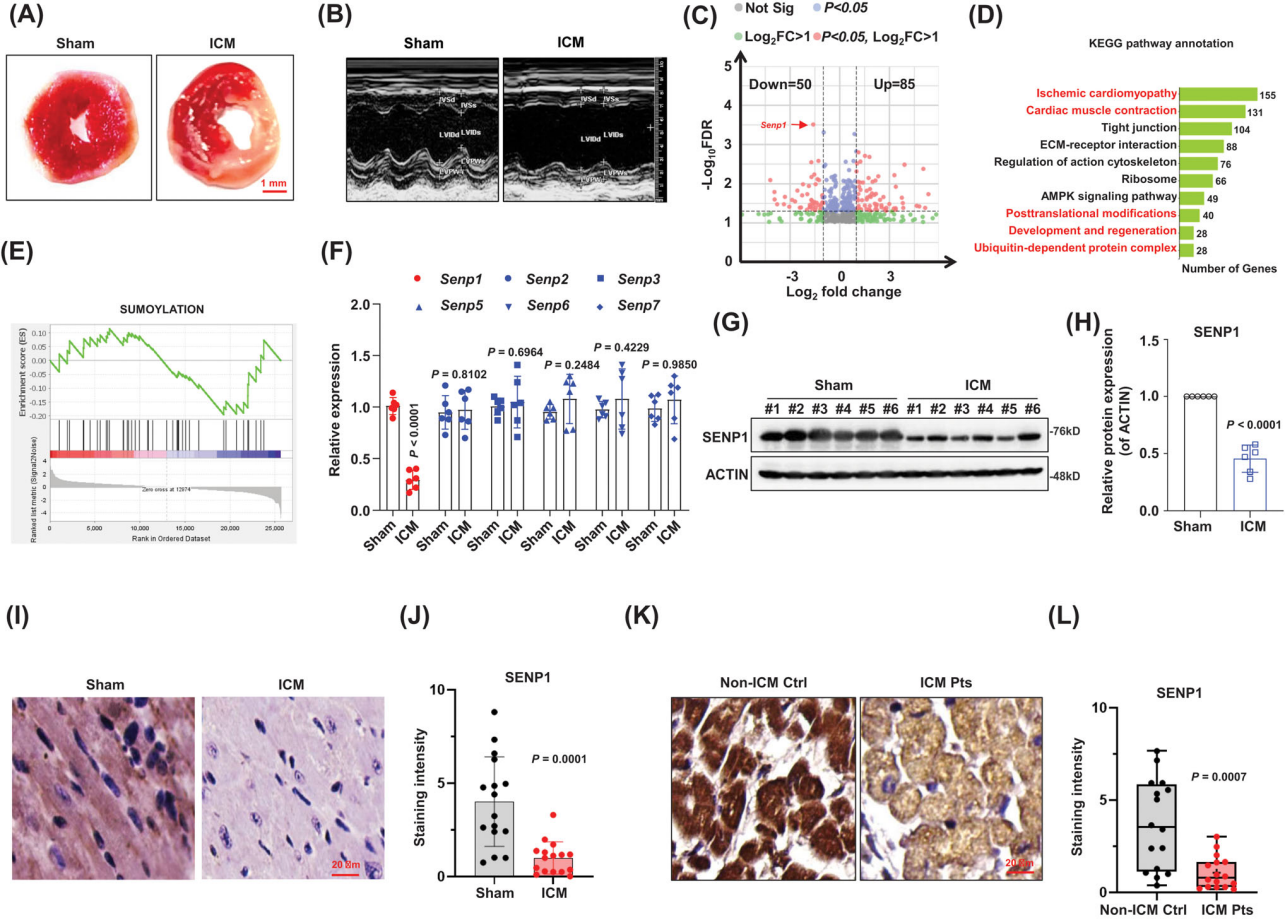
SENP1 is downregulated in ICM. (A) TTC staining of heart tissues from the ICM and sham mice (*n* = 6 mice per group). (B) Echocardiographic analysis of the structure of left ventricle (*n* = 6 mice per group). (C) Volcano plot showing differently expressed genes in the ICM tissues compared to the sham control (*n* = 3 sample from 3 independent mouse). (D) KEGG analysis revealing the top 10 enriched biogenesis of the DEGs. (E) GSEA analysis for downregulated gene in the ICM mice. (F) qPCR analysis of *Senp* family genes in the heart tissue of ICM compared to the controls (*n* = 6 sample from 6 independent mouse). (G) Western blot validating levels of SENP1 expression in the tissues from ICM or sham mice (*n* = 6 sample from 6 independent mouse), and quantification (H). (I) Representative images of IHC staining (SENP1) of heart tissue from ICM or sham mice, and (J) quantification for the staining intensity of SENP1 protein (*n* = 6 mice per group). (K) Immunostaining of SENP1 in heart tissue from ICM patients (ICM Pts) and non‐ICM controls (non‐ICM Ctrl), and (L) quantification for the staining intensity of SENP1 protein on at least 5 slide per patients (*n* = 3 patients per group). Data indicates mean ± SEM. Two‐sided *p* values were determined by Student's *t*‐test.

First, we successfully induced an ischemic cardiomyopathy (ICM) model in mice. The infarct volume was measured using 2,3,5‐triphenyl‐tetrazolium‐chloride (TTC), and the results demonstrated an obvious infarct area in ICM mice compared with the sham controls (Figures 1A and S1A), along with the significantly increased ratio of heart‐weight/body‐weight, reduced left ventricular ejection fraction (LVEF%), enlarged cardiomyocyte volume and aggravated fibrosis, as compared with the sham controls (Figure S1B–F). Echocardiographs were recorded during ischemia 2 weeks after surgery to evaluate cardiac contractile function, revealing a decrease in cardiac fractional shortening in ICM mice (Figures 1B and S1G–I). To identify differentially expressed genes related to ICM, we conducted RNA‐sequencing analysis on heart tissues at marginal zone of infarcted area. The analysis revealed 85 upregulated genes and 50 downregulated genes in ICM group compared to sham group (Figure 1C). KEGG enrichment analysis of the differentially expressed genes suggested their involvement in responses to ischemic cardiomyopathy, cardiac muscle contraction, and posttranslational modifications (Figure 1D). We also observed that some downregulated genes were enriched in the SUMOylation biological process (Figure 1E), including *Senp1*, a deSUMOylase specifically targets SUMO‐conjugated substrates, which is listed as one of the top differently expressed genes. To validate our RNA‐seq findings, we analysed expressions of the SENPs family through qPCR and western blotting. Among the *Senp* family genes, *Senp1* was the only gene found to be decreased in the hearts of ICM mice (Figure 1F). The reduced expression of SENP1 in the heart tissues of ICM mice compared to the sham group was confirmed (Figure 1G and H). Immunohistochemical and immunofluorescence analysis of myocardial tissue also showed decreased SENP1 expression in ICM mice (Figures 1I and 1J and S1J). Furthermore, to investigate the clinical relevance of our findings, we analysed SENP1 expression in cardiac tissues from patients with ICM and non‐ICM controls. The results (Figure 1K and L) indicated a decreased expression of SENP1 in patient samples, suggesting a correlation between SENP1 and ICM. These collective findings support the association of SENP1 with ICM.

### 3.2 Knockdown of SENP1 facilitates hypoxia‐induced apoptosis

**FIGURE 2:**
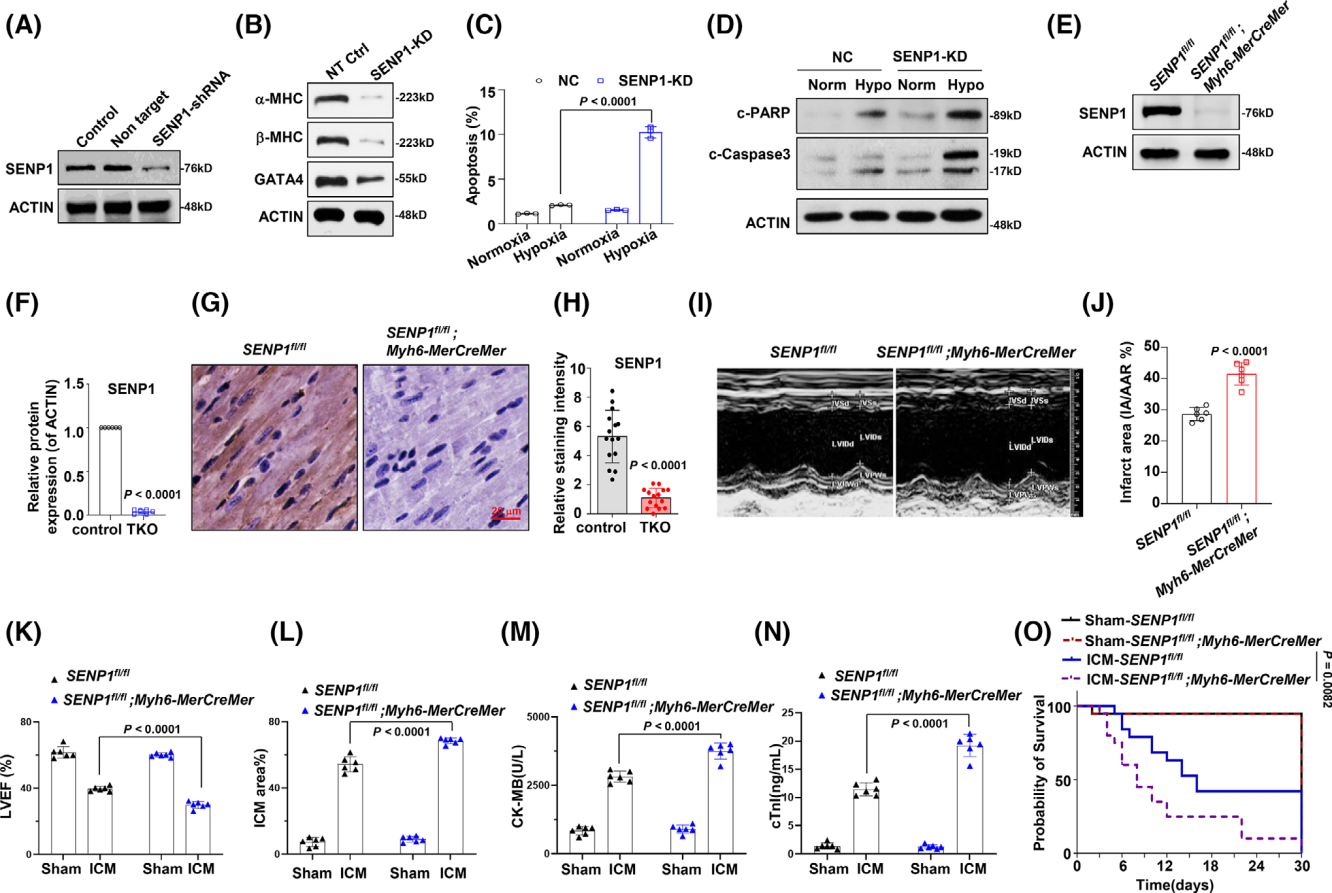
Suppression of SENP1 deteriorates ICM. (A)Western blotting showing efficiency of SENP1 suppression using lentivirus carrying shRNAs in H9c2 cells. (B) Western blot depicting levels of α‐MHC, β‐MHC and GATA4 in H9c2 cells after SENP1 knockdown. (C) Statistical analysis of the apoptotic H9c2 cells after SENP1 knockdown under hypoxic condition (*n* = 3 each group). (D) Western blotting showing c‐PARP and c‐Caspase3 levels in H9c2 cell after SENP1 knockdown under hypoxia condition. (E) Western blotting showing SENP1 levels in the heart tissues from *Senp1* ^fl/fl^ and *Senp1* ^fl/fl^; *Myh6‐MerCreMer* (*Senp1*‐TKO) mice, and quantification (F) (*n* = 6). (G) Immunohistochemistry staining of SENP1 in heart tissue from control mice and *Senp1*‐TKO mice, and quantification (H). (I) Representative M‐mode echocardiographic images for control mice and *Senp1*‐TKO mice under ICM conditions (*n* = 6/group). (J) Infarct area (IA/AAR) of control mice and TKO mice under ICM conditions (*n* = 6/group). (K) Left ventricular ejection fraction (LVEF) of control mice and TKO mice under ICM conditions (*n* = 6/group). (L) ICM area percentage, (M) Creatine Kinase MB (CK‐MB) level, (N) Cardiac Troponin I (cTnI) level in the control mice and TKO mice under ICM conditions (*n* = 6/group). (O) Survival rate of the control mice and TKO mice of ICM groups (*n* = 12/group). All the ICM groups were compared with the sham groups. Data indicates mean ± SEM. Two‐sided *p* values were determined by Student's *t*‐test.

To further elucidate the role of SENP1 in regulating cardiomyocyte function, we initially transduced H9c2 cells with lentivirus expressing shRNA targeting SENP1 (Figures 2A and S2A). Our findings revealed that knockdown of SENP1 led to the suppression of key proteins essential for heart function, including alpha‐myosin heavy chain (α‐MHC), β‐MHC, and GATA Binding Protein 4 (GATA4) (Figures 2B and S2B). Given that apoptosis often accompanies hypoxia and myocardial infarction progression, we subsequently assessed the apoptosis of SENP1‐knockdown (KD) and control cells under hypoxic conditions. Interestingly, our results demonstrated that SENP1 depletion exacerbated hypoxia‐induced myocyte apoptosis, as evidenced by markedly augmented apoptotic cell rates and cleaved PARP and Caspase‐3 (Figures 2C and D and S2C and D). To investigate the impact of SENP1 in myocardial ischemia in vivo, we generated cardiac muscle tissue‐specific SENP1 knockdown (SENP1‐TKO) mice by crossing *Myh6*‐*MerCreMer* mice with *SENP1* ^fl/fl^ mice.^21^ Subsequent analysis confirmed reduced SENP1 expression in TKO mice (Figure 2E–H). Echocardiographic assessment revealed impaired cardiac function in TKO mice following ischemic cardiomyopathy (ICM) injury compared to controls (Figures 2I and S2E). Additionally, considerably increase infarcted area in ICM mice (Figures 2J and S2F), reduced left ventricular ejection fraction (LVEF%) (Figure 2K), decreased fractional shortening (LVFS%) (Figure S2G), increased myocardial infarction area percentage, elevated Creatine Kinase MB (CK‐MB), and cardiac troponin levels were observed in SENP1‐TKO mice post‐ICM (Figure 2L–N). Moreover, the survival probability of TKO mice post‐ICM was lower compared to control mice, collectively highlighting the critical role of SENP1 in preserving cardiac function in the context of ICM (Figure 2O).

### 3.3 SENP1 interacts and stabilises MEF2C

**FIGURE 3:**
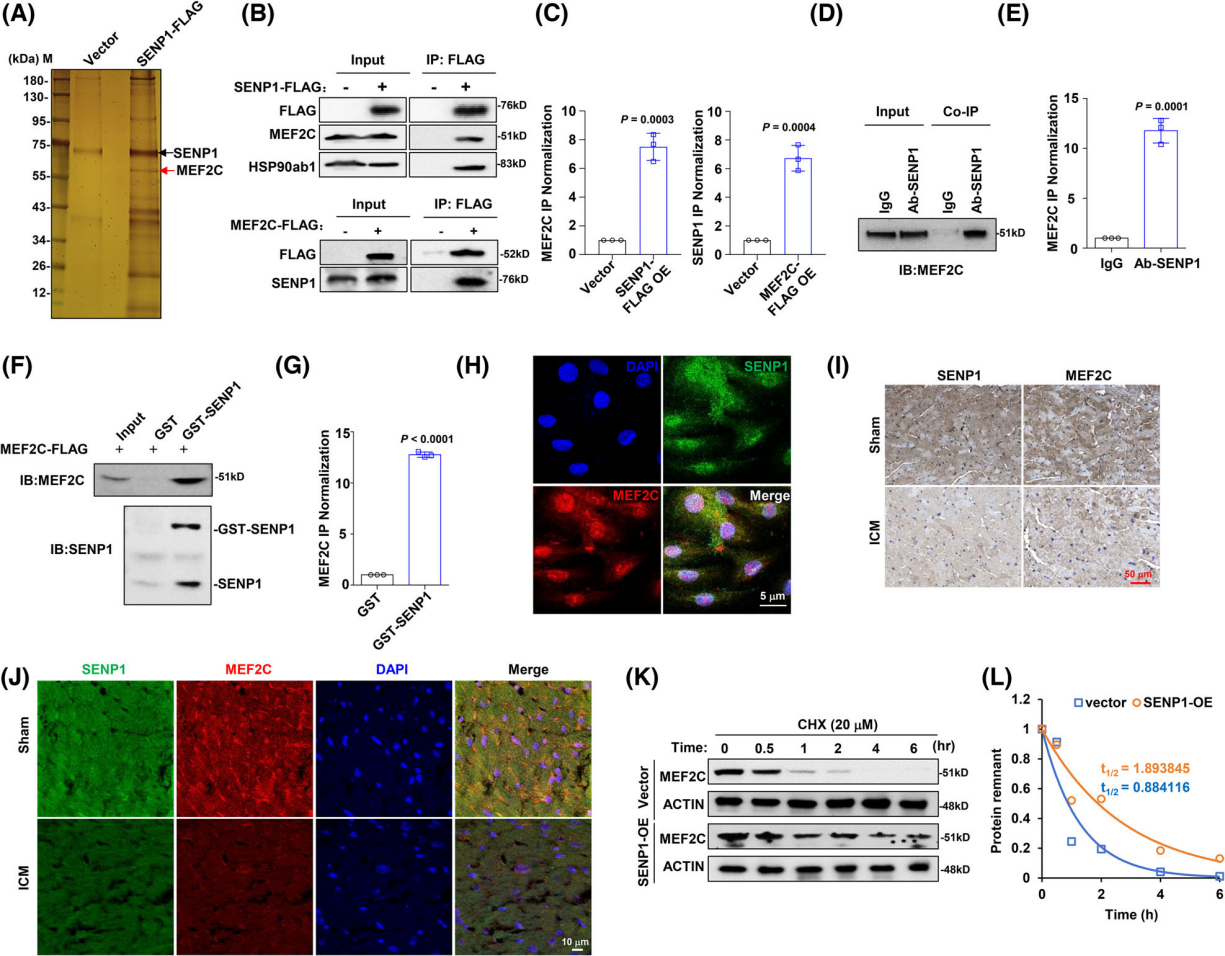
SENP1 interacts and stabilises MEF2C protein. (A) Silver staining illustrating FLAG‐pulldown of FLAG‐SENP1 complex in HEK293T cells. (B) Co‐IP assay validating the interaction between exogenous FLAG‐ SENP1 with MEF2C (upper panel), and FLAG‐MEF2C with SENP1 in HEK293T cells (lower panel), and (C) quantification of the Co‐IP MEF2C or SENP1 to their negative controls (*n* = 3), respectively. (D) Endogenous interaction of MEF2C with SENP1 in H9c2 cells. Input indicates 2% whole cell lysate. Ab‐SENP1 denotes, anti‐SENP1 antibody. (E) Quantification of Co‐IP for SENP1 to the IgG control (*n* = 3). (F) GST‐pulldown assay showing the direct interaction between purified MEF2C‐FLAG and GST‐tagged‐SENP1 protein in vitro. (G) Quantification of GST‐SENP1 compared to the GST control (*n* = 3). (H) Confocal fluorescence images depicting the co‐localisation of MEF2C and SENP1 in H9c2 cells. (I) Immunohistochemistry staining for SENP1 and MEF2C protein levels in serial sections of heart tissues from ICM model and sham group. Scale bar, 50 µm. (J) Immunofluorescence staining for SENP1 and MEF2C protein levels in serial sections of heart tissues from ICM model and sham group. Scale bar, 10 µm. (K) Degradation rate of MEF2C protein in H9c2 cells co‐expressed with SENP1 or vector control in presence of 20 µM cycloheximide (CHX) for up to 6 h. (K) The calculated half‐life of MEF2C protein in the above H9c2 cells. Data indicates mean ± SEM. Two‐sided *p* values were determined by Student's *t*‐test.

The deconjugating enzyme SENP is an important regulator of cellular events under physiological and pathophysiological conditions. To further investigate how dysregulated SENP influences myocardial infarction, we artificially overexpressed FLAG‐tagged SENP1 in HEK293T cells and immunoprecipitated the SENP1 complex for mass spectrometry (Table S2). In this analysis, we identified a protein within the complex, namely MEF2C, which has been discovered as a key regulator for physiological and pathophysiological processes of cardiomyocytes^22^ (Figure 3A). Subsequently, when we immunoprecipitated the MEF2C or SENP1 protein respectively to determine the protein‐protein interaction, we found that they could indeed bind to each other (Figure 3B and C). Moreover, an obvious endogenous interaction was also observed in H9c2 cells (Figure 3D and E). We subsequently generated and purified exogenous GST‐fusion SENP1 protein and MEF2C‐FLAG fusion protein, confirming their direct interaction in vitro (Figure 3F and G). Through immunofluorescence staining, we found that MEF2C and SENP1 co‐localise in the nucleus (Figure 3H). Immunohistochemistry and immunofluorescence staining showed that expressions of SENP1 and MEF2C were accordant in the ICM mice model compared to the sham group (Figure 3I and J). Importantly, SENP1 exhibited a protective effect on the stability of MEF2C, as the half‐life of MEF2C was significantly extended in the presence of overexpressed SENP1 (Figure 3K and L).

### 3.4 SUMO2‐mediated SUMOylation at K401 promotes MEF2C protein stability

**FIGURE 4:**
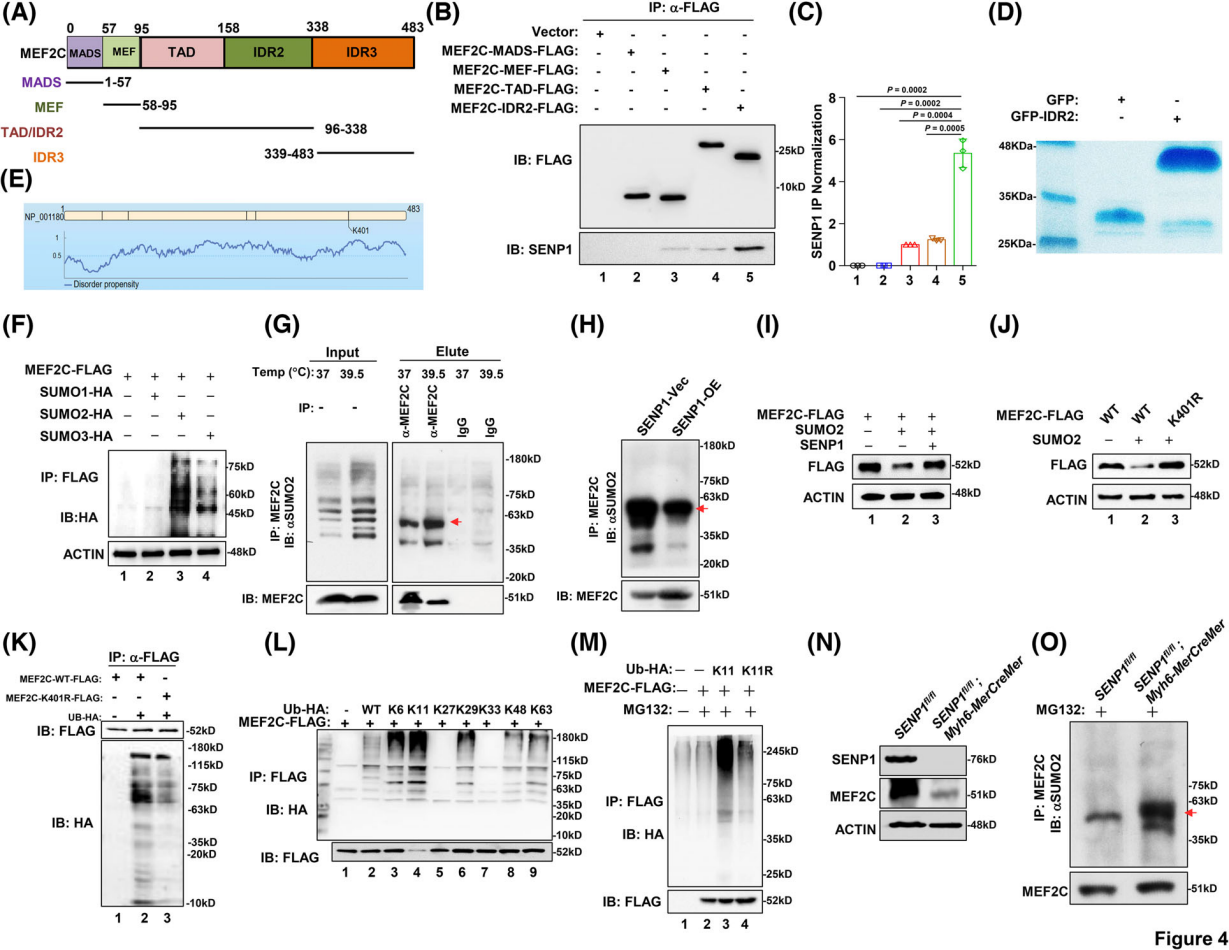
SUMOylation modification of MEF2C affects ubiquitination‐linked protein degradation. (A) Mapping of MEF2C protein structures. (B) FLAG pull‐down assay showing interacting domains of MEF2C and SENP1 in HEK293T cells, and quantification (C). (D) Coomassie blue staining shows in vitro expression of GFP‐fusion IDR2 protein or GFP protein. (E) Prediction of SUMOylation site of MEF2C. (F) Co‐IP assay demonstrating the interactions between MEF2C‐FLAG with SUMO1/2/3‐HA. (G) Endogenous Co‐IP assay demonstrating SUMO2‐conjugated SUMOylation of MEF2C protein. (H) SUMO2‐conjugated SUMOylation level of MEF2C in HEK293T cells overexpressing SENP1. (I) Western blotting showing MEF2C levels in HEK293T cells after MEF2C and SUMO2 or SENP1 overexpression. (J) Exogenous level of FLAG‐tagged MEF2C^Wt^ or MEF2C^K401R^ co‐expressing with SUMO2 in HEK293T cells. (K) Ubiquitination levels of FLAG‐tagged MEF2C^Wt^ or MEF2C^K401R^ in HEK293T cells. (L) Co‐IP assay illustrating the interacting types of ubiquitin and MEF2C protein. K6, ubiquitin only contains lysine residue at position 6, others were mutated. (M) Co‐IP assay validating the K11‐ubiquitin conjugated ubiquitination of MEF2C protein. K11R, ubiquitin contains all lysine residues except the lysine residue at position 11 was mutated. (N) Western blotting showing SENP1 and MEF2C levels in the heart tissues from *Senp1* ^fl/fl^ and *Senp1* ^fl/fl^; *Myh6‐MerCreMer* (*Senp1*‐TKO) mice. (O) SUMO2‐conjugated SUMOylation level of MEF2C in the heart tissues from *Senp1* ^fl/fl^ and *Senp1* ^fl/fl^; *Myh6‐MerCreMer* (*Senp1*‐TKO) mice. Data indicates mean ± SEM. Two‐sided *p* values were determined by Student's *t*‐test.

To determine the mechanism underlying SENP1‐mediated stabilisation of MEF2C, we designed truncated versions of MEF2C (Figure 4A) for FLAG‐pulldown experiments. Upon expression and subsequent FLAG‐immunoprecipitation of these truncations, we observed that SENP1 primarily interacts with the MEF2C‐IDR2 domain (Figure 4B and C). We purified exogenous GFP‐IDR2 protein for further analysis (Figure 4D). Given that SENP1 functions as a desumoylating enzyme, the prediction of GPS‐SUMO website shows that residue K401, serves as a potential site for SUMOylation (Figure 4E). Subsequently, we co‐expressed MEF2C‐FLAG with SUMO1‐HA, SUMO2‐HA, or SUMO3‐HA overexpression plasmids in HEK293T cells, establishing that the interaction between MEF2C and SUMO2 predominates (Figures 4F and S3A). Indeed, SUMO2‐mediated SUMOylation of endogenous MEF2C protein could be detected in a Co‐IP assay (Figure 4G), and the SUMOylation level was markedly attenuated when SENP1 was forcedly expressed in HEK293 cells (Figure 4H). It is shown that MEF2C could be SUMOylated by SUMO2, associated with the subsequent ubiquitination degradation, and overexpression of SENP1 attenuated the ubiquitination of MEF2C (Figures 4I and S3B and C). To elucidate the impact of K401‐linked SUMOylation on MEF2C degradation, we generated a K401R mutation, leading to a marked increase in MEF2C protein expression (Figures 4J and S3D and E). Through immunoprecipitation (IP) assays, we also determined that K401‐linked SUMOylation was correlated with MEF2C ubiquitination (Figures 4K and S3F). Further experimentation with a range of ubiquitination plasmids revealed that MEF2C ubiquitination is predominantly mediated by K11 (Figures 4L and S3G); upon mutation of K11, polyubiquitination of MEF2C was markedly reduced (Figures 4 M and S3H and I). Importantly, in the SENP1‐CKO cardiomyocyte, the MEF2C protein was correspondingly suppressed (Figure 4N), along with augmented SUMOylation level of MEF2C (Figure 4O). Consequently, these findings suggest that MEF2C protein degradation primarily occurs through K11‐linked ubiquitination.

### 3.5 MEF2C is a phase separation forming protein

**FIGURE 5:**
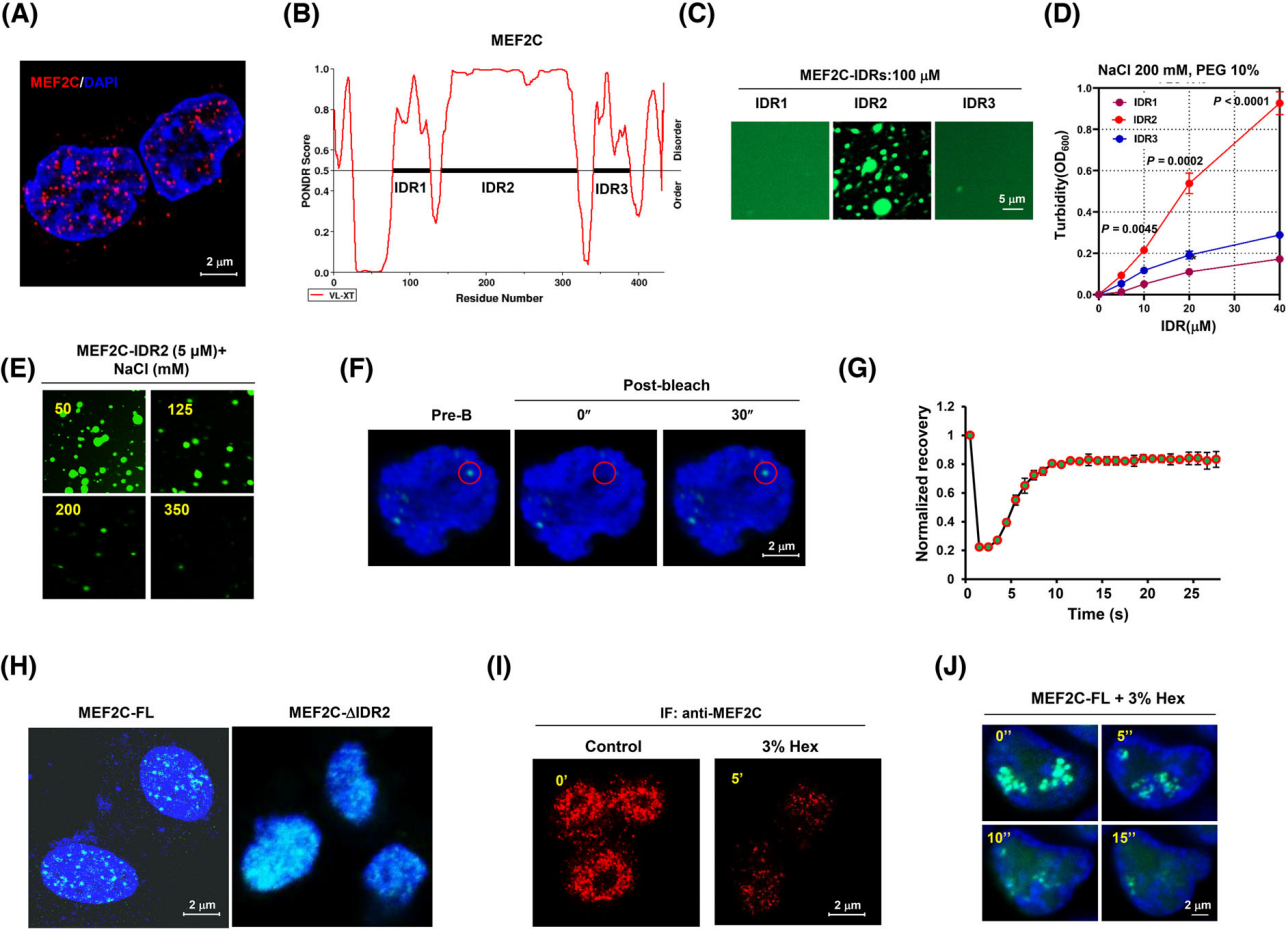
MEF2C is a nuclear condensation forming protein. (A) Representative IF images for endogenous foci of MEF2C in H9c2 cells. (B) Prediction of IDRs of MEF2C protein using PONDR algorithms. (C) Visualisation of droplet formation in vitro for the GFP‐tagged MEF2C‐IDRs. (D) Turbidity assay (OD600) of GFP‐tagged MEF2C‐IDRs in 200 mM NaCl and 10% PEG. (E) Representative images of droplet formation of GFP‐IDRs at different salt concentration (*n* = 3 independent experiments). (F) Fluorescence recovery after 5″ photobleaching (FRAP) of MEF2C‐GFP focus (red framed) by 488 nm laser and 30″ recovery (*n* = 3 independent experiments). (G) Kinetic recovery times of bleached GFP‐tagged MEF2C foci (*n* = 3 independent experiments). (H) Phase separation formation of full‐length MEF2C (FL) and IDR2 depleted MEF2C‐ΔIDR2 in HEK293T cells. Scale bar, 2 µm. (I) Endogenous MEF2C foci in H9c2 cells before and after treatment with 3% 1,6‐hexanediol for 5 s. (J) Exogenous MEF2C‐FLAG foci in H9c2 cells before and after treatment with 3% 1,6‐hexanediol for 5 s. (*n* = 3 biologically independent experiments). Data indicates mean ± SEM. Two‐sided *p* values were determined by Student's *t*‐test.

In our immunofluorescence analysis, we observed that endogenous MEF2C protein was present as distinct puncta rather than being evenly distributed in H9c2 cells, suggesting a possible phase separation (Figure 5A). Nuclear compartmentalisation has recently been recognised as a mechanism for the quantitative control of gene expression and cell function.^23^ We then sought to determine whether MEF2C could undergo phase separation. Predicting the MEF2C protein structure, we identified three putative intrinsically disordered regions (IDRs) (Figure 5B). To evaluate the importance of the IDR region for MEF2C phase separation, we constructed GFP‐fused IDR1, IDR2, and IDR3 to measure their droplet formation in vitro and found that only GFP‐IDR2 formed droplets at a concentration of 100 µM (Figure 5C). The turbidity assay also showed that IDR2 exhibited higher turbidity than the other IDR fusion proteins (Figure 5D). Furthermore, to further verify the phase separation property of MEF2C, we added purified MEF2C‐IDR2 at increasing concentrations of NaCl and found a significantly decreased phase separation formation at higher NaCl concentrations (Figure 5E). Additionally, similar puncta were observed in live H9c2 cells when expressing the GFP‐MEF2C fusion protein. Upon bleaching with a 488‐nm laser, the bleached puncta quickly reassembled (Figure 5F, red‐framed foci), and the kinetic recovery of GFP‐MEF2C fluorescence showed that most of the foci fluorescence recovered within 15 s (Figure 5G). To further evaluate the significance of the IDR region in MEF2C phase separation, we created GFP‐tagged full‐length (FL) protein and a GFP‐fusion protein lacking the IDR2 domain to assess droplet formation. Interestingly, we observed that only the MEF2C‐FL could aggregate, while the removal of the IDR2 domain led to a more dispersed distribution of MEF2C (Figure 5H). Finally, we found that the treatment of 1,6‐hexanediol caused a reduction in the formation of endogenous MEF2C puncta (Figure 5I), and this outcome was similarly observed in exogenously expressed GFP‐MEF2C puncta in H9c2 cells (Figure 5J). These findings collectively indicated that MEF2C is a protein possessing phase separation forming property.

### 3.6 In presence of SENP1 enhances formation of nuclear condensation of MEF2C

**FIGURE 6:**
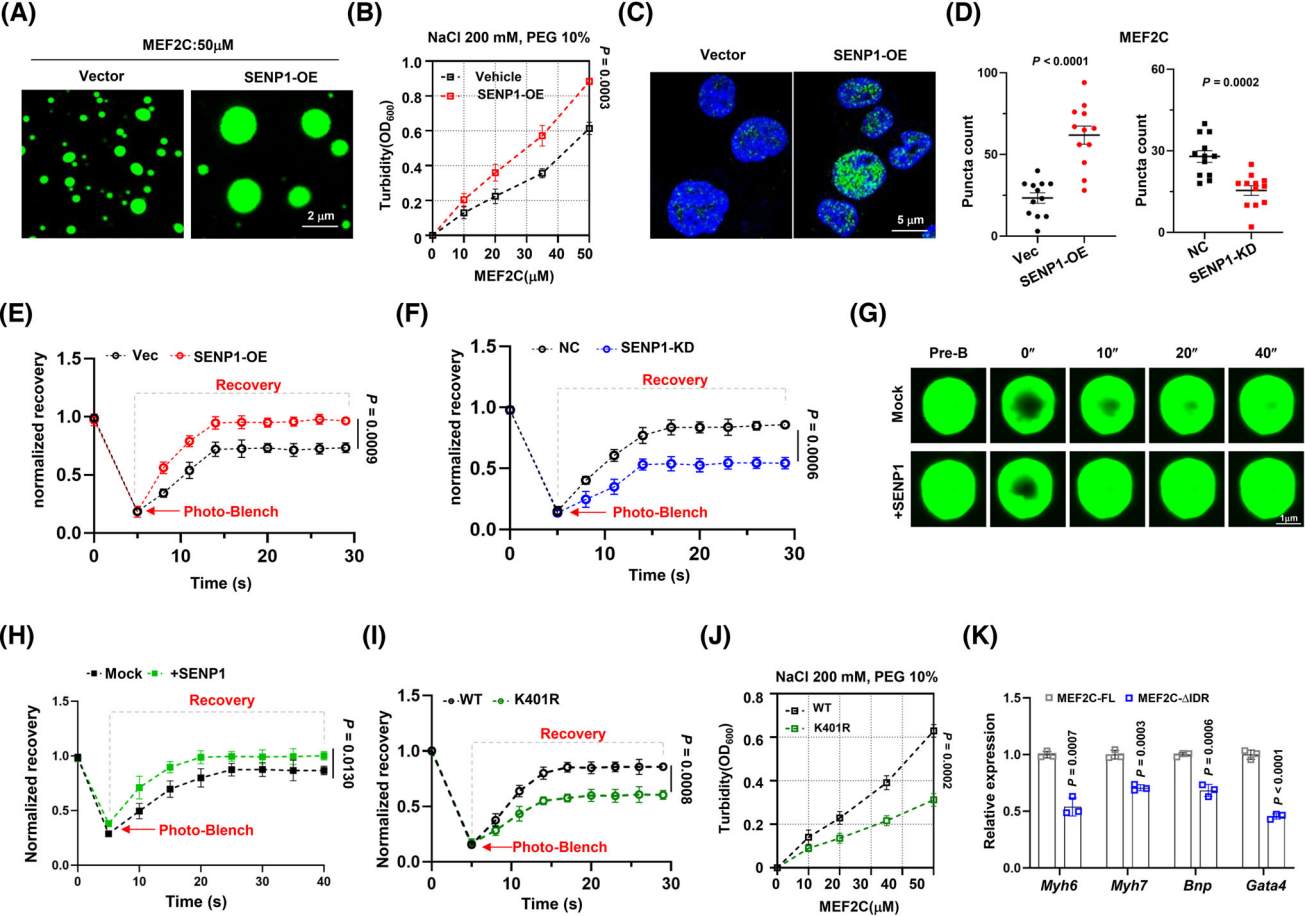
SENP1 promotes nuclear condensation of MEF2C. (A) In vitro droplet formation of MEF2C‐GFP in the presence or absence of SENP1. (B) Turbidity assay (OD600) of MEF2C‐GFP in the presence or absence of SENP1 in 200 mM NaCl and 10% PEG. (*n* = 3 biologically independent experiments). (C) Confocal fluorescence images of MEF2C puncta in the nucleus of H9c2 cells. (Scale bars: 5 µm) (D) Statistical analysis of MEF2C puncta counts in H9c2 cells with SENP1 overexpression or knockdown (*n* = 3 biologically independent experiments). (E, F) Kinetic recovery times of bleached GFP‐tagged MEF2C droplet foci in SENP1 overexpressing or SENP1 knockdown H9c2 cells. (*n* = 3 biologically independent experiments). (G) FRAP assay of GFP‐tagged MEF2C droplet photobleaching with or without SENP1 in vitro. (H) Kinetic recovery times of bleached GFP‐tagged MEF2C droplet in vitro. (I) Kinetic recovery times of bleached GFP‐tagged WT or K401R MEF2C droplet in H9c2 cells. (J) Turbidity assay (OD600) of GFP‐tagged WT or K401R MEF2C droplet in 200 mM NaCl and 10% PEG. (*n* = 3 biologically independent experiments). (K) qPCR analysis of expressions of targeted genes in the MEF2C‐FL or MEF2C‐∆IDR overexpressing H9c2 cells. (*n* = 3 biologically independent experiments). Data indicates mean ± SEM. Two‐sided *p* values were determined by Student's *t*‐test.

The formation and dissolution of condensates are intricately controlled within the intracellular milieu. Inadequate regulation of condensate properties can result in protein misfolding and aggregation, contributing to the pathogenesis of numerous diseases.^24^ Therefore, we sought to investigate the regulatory role of SENP1 on MEF2C condensate assembly and heart function. Our findings indicated a pronounced increase in the formation of MEF2C in vitro in the presence of SENP1 (Figure 6A), and the turbidity assay showed higher turbidity of MEF2C in the presence of SENP1 (Figure 6B). Furthermore, ectopic overexpression of SENP1 was observed to potentially promote the formation of MEF2C condensation in H9c2 cells (Figure 6C and D). In vivo, when the GFP‐MEF2C foci were bleached by a 488 nm laser, the kinetic recovery of GFP‐MEF2C fluorescence was significantly faster when SENP1 was overexpressed than that in cells with SENP1 suppression (Figure 6E and F). In vitro, when the droplets of MEF2C‐GFP fusion protein were accompanied by SENP1‐FLAG protein, the fluorescence recovery was markedly accelerated compared to the vector control (Figure 6G and H). However, when the K401 site was mutated to arginine, the fluorescence recovery of MEF2C‐GFP protein expressed in H9c2 cells was significantly suppressed compared with the wild‐type control (Figure 6I); meanwhile, the turbidity of the MEF2C‐K401R abated evidently compared to the wild‐type control at the same concentration of NaCl (Figure 6J). When the MEF2C‐∆IDR vector was forcibly expressed in H9c2 cells, the expressions of genes critical for heart function were all prominently suppressed because of the domain‐negative effect (Figure 6K). These findings suggest that condensation formation of MEF2C will be enhanced when SENP1 is in presence, and highlight the importance of MEF2C condensation formation for cardiac function.

### 3.7 Cardiac rescue of SENP1 alleviates ischemic heart injury in mouse model

**FIGURE 7:**
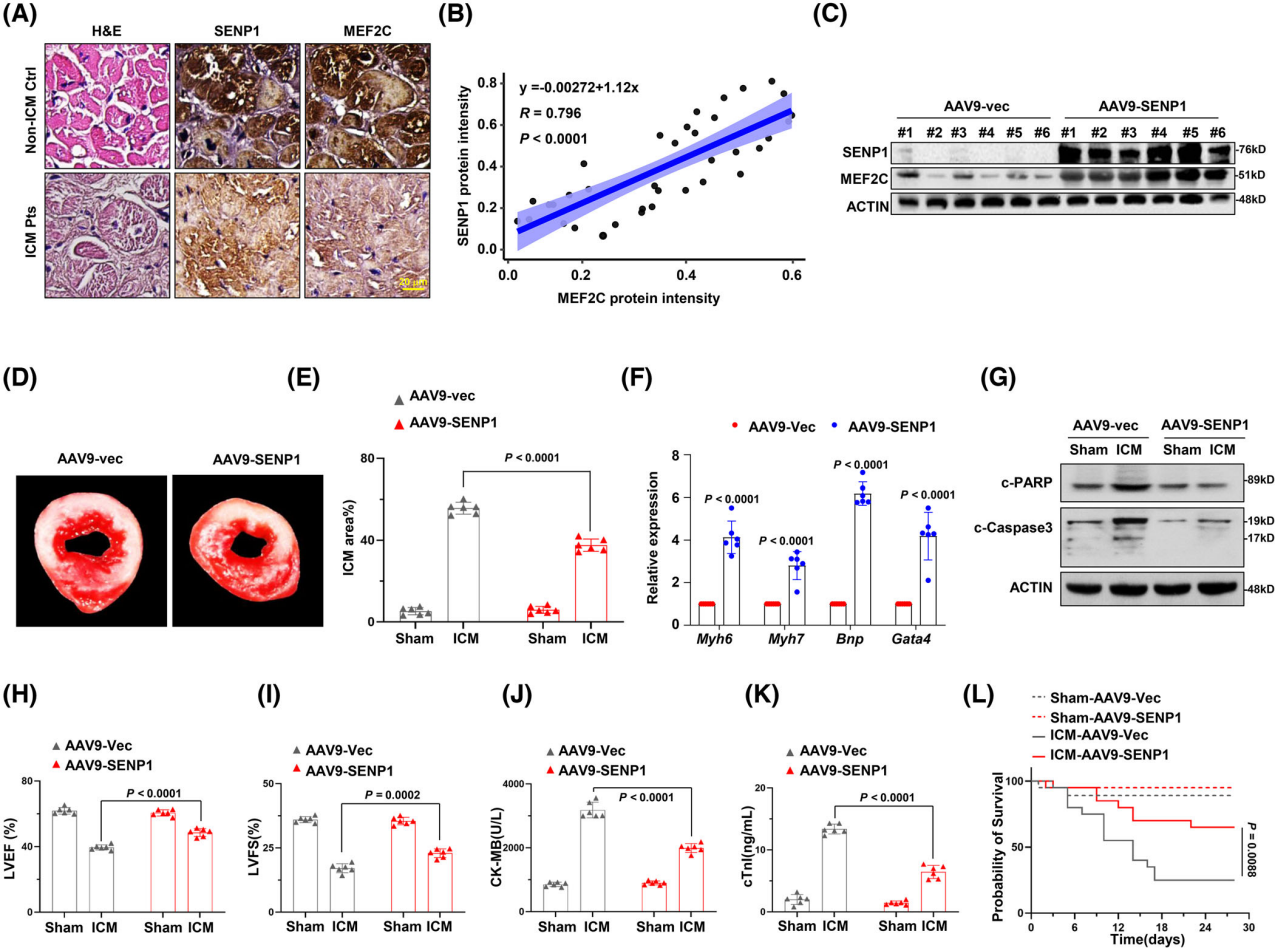
Rescue of SENP1 ameliorates ICM in vivo. (A) Immunohistochemistry staining for SENP1 and MEF2C protein levels and HE staining in heart tissues from non‐ICM controls (non‐ICM Ctrl) and ICM patients (ICM Pts). Scale bar, 20µm. (B) Correlation of SENP1 and MEF2C protein according to IHC intensity in heart tissues from ICM patients of (A). (C) Western blotting showing SENP1 and MEF2C levels in 6 representative mice infected with AAV9‐SENP1 or AAV9‐vector control. (D) Representative photographs of TTC staining for heart tissues from ICM mice infected with AAV9‐SENP1 or AAV9‐vector. (E) ICM area of the above‐mentioned mice (*n* = 6/group). (F) qRT‐PCR analysis for expression of target genes in the AAV9‐SENP1 and AAV9‐vector mice (*n* = 6/group). (G) Western blotting showing c‐PARP and c‐Caspase3 levels in heart tissues from ICM mice infected with AAV9‐SENP1 or AAV9‐vector. (H) Percentage of left ventricular ejection fraction (LVEF), (I) Percentage of left ventricular fractional shortening (LVFS), and (J) Creatine Kinase MB (CK‐MB) levels, (K) Cardiac Troponin levels (cTnI) in the AAV9‐Vec or AAV9‐SENP1 treated mice 4 weeks after being subjected to ICM (*n* = 6/group). (L) Survival rate of the AAV9‐Vec or AAV9‐SENP1 infected ICM mice (*n* = 12/group). Data indicates mean ± SEM. Two‐sided *p* values were determined by Student's *t*‐test.

Having demonstrated the critical role of SENP1 in stabilising MEF2C and protecting heart under conditions of ischemic injury, we seek to elucidate the significance of SENP1 in the clinic and its translational potential. We analysed the protein levels of SENP1 and MEF2C in heart tissues from non‐ICM controls and patients with ICM, and found a significant correlation between these two proteins (Figure 7A and B). In mice, when SENP1 was successfully delivered by adeno‐associated virus type 9 (AAV‐9) to generate the AAV9‐SENP1 and AAV9‐mock mice for the ICM model without affecting *Mef2c* mRNA level (Figures 7C and S4A and B), we observed that the AAV9‐SENP1‐treated group had less severe injury compared to the AAV9‐mock group (Figure 7D and E). Accordingly, genes critical for heart function were also significantly increased in the heart tissues of AAV9‐SENP1 mice (Figure 7F), and apoptosis of cardiomyocytes were markedly alleviated, as shown by the reduction in cleaved PARP and Caspase 3(Figures 7G and S4C). LVEF and LVFS were distinctly improved (Figure 7H and I), accompanied by decreased CK‐MB and cTnI levels (Figure 7J and K). Furthermore, the mortality rate was reduced to about 20% in the AAV9‐SENP1‐treated mice (Figure 7L). These results imply the protective role of SENP1 in ischemic injury in mice.

## 4 DISCUSSION

Understanding the intracellular signalling of cardiomyocytes in response to ischemic injury is crucial for preventing of progression into heart failure and holds promise for exploiting these mechanisms for therapeutic use. To date, many processes remain poorly understood, impeding the advancement of new treatment approaches. In this study, we used RNA‐seq to analyse significantly changed genes in cardiomyocytes after ischemic damage and identified an important role of *Senp1* in protecting cardiomyocytes from ischemic injury both in vivo and in vitro. We propose a new mechanism in which SENP1 participates SUMOylation modification and protein stability of MEF2C, meanwhile, SENP1 interacts with MEF2C and promotes its nuclear condensation to favour the expression of genes essential for cardiac function and cardiomyocyte viability. Translating our findings, our data suggests that using AAV9‐mediated Senp1 overexpression could alleviate the progression of a murine model of ischemic cardiomyopathy (ICM), offering promise for treating patients suffering from ischemic heart disease.

SUMOylation and deSUMOylation coordinately affect protein levels and thereby play important roles in the regulation of multiple cellular signalling pathways in cardiac functions and development, specifically ICM injury.^25^ Previous work has indicated that the regulation of the extent of SUMOylation of some proteins determines the response and fate of cardiomyocytes after cardiac insults,^26^ thus either alleviating or exacerbating myocardial damage.^27^ SENP2 functions as a deSUMOylating enzyme for GATA4 and various other transcription factors that are essential for cardiac development.^25^ Through RNA‐seq analysis, we identified decreased SENP1 expression upon ischemic stimulation in cardiomyocytes. By analysing the proteins that interact with SENP1, we successfully identified the transcription factor MEF2C as relevant for severe heart ischemic injury in TKO mice. MEF2C belongs to the MEF2 transcription factor family, playing an early and prominent role in cardiovascular development and differentiation.^28^ However, dissecting the specific roles of MEF2C in cardiac injury is complicated by its unique regulatory activities.^29^ SUMOylation occurs at specific sites on target proteins, and identifying these SUMOylation sites holds promise for the development of new drugs. Dysfunction of transcription factors has been identified as associated with the response to ischemic stress and the progression to heart failure. We have uncovered a novel molecular mechanism for ICM and established an association between SENP1 and MEF2C stability. Our study reveals that SENP1 mediates the deSUMOylation of MEF2C at the K401 site for stabilising protein stability. We propose that SUMOylation modification of MEF2C is dominantly mediated by SUMO2, however, further investigations are needed to clarify the effect of SUMOylation. These findings correspond to characteristics observed in a mouse model. By combining our present findings on SENP1 and MEF2C deSUMOylation in protecting against ICM, it is highly likely that the modulation of the SUMO system for a selective transcription factor within cardiomyocytes is critical in the pathogenesis of cardiac disease. However, further investigations are warranted to determine whether SENP1 is involved in other heart cells.

Phase separation is recognised to contribute to various cellular processes, such as the formation of classical membraneless organelles, signalling complexes, the cytoskeleton, and many other supramolecular structures. Emerging evidence indicates that condensates of proteins induce the formation of membrane‐less organelles, exhibiting higher protein density and allowing for increased rates of biochemical reactions.^30^ Abnormal LLPS is involved in various biological activities and disease conditions.^31^ Wada H et al revealed that changes of expression and activity of regulatory proteins, such as PIASx, SENP1, SENP2, and TRIM11, modulated the aggregation of tau proteins.^32^ Our study reveals that the interaction with SENP1 controls the nuclear condensation of MEF2C to promote the expression of genes critical for cardiomyocyte function. Thus, overexpression of SENP1 by manipulating gene expression promotes droplet formation and protein stability of MEF2C, thereby enabling resistance to ischemic injury. Our study also suggests that phase separation of MEF2C is necessary for exerting its biological function as a transcriptional factor in reprogramming expression profile of cardiomyocyte, since abolishment of phase separation impairs expressions of key genes governing cardiac remodelling, such as *Myh6, Myh7, Bnp*, and *Gata4*. Although, our study suggests that SENP1 promotes stability and condensation of MEF2C, but we can't clarify the causal relation between protein stability and condensation, which need further investigations. Collectively, the findings of this study provide new evidence of the interaction with SENP1 promoting LLPS in cells, especially in cardiac disease, solving a puzzle regarding the regulation mechanism of MEF2C stability.

Translationally, we evaluated the effect of overexpressing SENP1 using AAV9 to overcome ICM in a mouse model. Our results provided strong evidence that SENP1 attenuates pathological cardiac failure in the mouse ICM model and improves left ventricular (LV) function. Several physical methods and synthetic small molecules are under development to target the SUMO pathway for the treatment of ICM. Our study updates the current understanding of SUMOylation in ICM and provides a theoretical basis for exploring SUMOylation‐targeted strategies as new therapies against ICM.

## AUTHOR CONTRIBUTIONS

YLW conceptualised the study, designed the experiments and wrote the original manuscript. YX performed the experiments, analysed the data. XQ and XYB helped with the animal experiments and plasmid constructions. QYL, YY and ZL performed data analysis. XL and TZ dealt with patient samples. XZL conceptualised and supervised the study.

## CONFLICT OF INTEREST STATEMENT

The authors declare no conflict of interest.

## Supporting information



Supporting Information

Supporting Information

Supporting Information

Supporting Information

## Data Availability

The RNA‐seq data can be found publicly in the Gene Expression Omnibus database under accession number GSE284743. All data are available in the main text or the supplementary materials, and are available from the corresponding author upon reasonable request.
