# Supplementary material for: Downregulation of SENP1 impairs nuclear condensation of MEF2C and deteriorates ischemic cardiomyopathy
Source: Clin Transl Med. 2025 May 7;15(5):e70318. doi: 10.1002/ctm2.70318 (PMC12059206; doi:10.1002/ctm2.70318)
Supplement: Supplementary file 1 — Supporting Information [file CTM2-15-e70318-s002.docx]

**Methods and Materials**

**Cell culture**

Rat embryonic cardiomyoblasts (H9c2 cell line) and HEK 293T cell were sourced from the Institute of Basic Medical Sciences, Chinese Academy of Medical Sciences in Beijing, China. The cells were nurtured in Dulbecco's Modified Eagle's Medium (Gibco, Carlsbad, CA, USA) supplemented with 10% FBS, 100 U/mL penicillin, and 100 μg/mL streptomycin (Hyclone, Logan, UT, USA) at 37°C with 5% CO_2_. For hypoxia induction, the cells were subjected to a hypoxic environment with 1% O2, 5% CO2, and 94% N2.

**Transfection, virus package and infection**

Transient transfections of HEK 293T cells were carried out using polyethyleneimine (PEI) from Polysciences in Warrington, PA, USA, in OPTI-MEM medium (Life Technologies, Carlsbad, CA, USA) with a DNA: PEI ratio ranging from 1:4 to 1:6.

Viral particles were generated by HEK 293T cells in a 10 cm dish transfected with 4 μg pMD2.G and 6 μg psPAX2 packaging plasmids (from Addgene, Watertown, MA, USA), along with 8 μg lentiviral vectors encoding target genes. The supernatant containing the viral particles was collected at 35 hr and 60 hr post-transfection, concentrated to 100 times its original volume using Poly (ethylene glycol) 8,000 from Sigma-Aldrich in St. Louis, MS, USA.

For viral infection, 1×10^6^ cells were seeded in 1 mL fresh complete media for 6 hours, then treated with 50 μL of viral concentrate and 8 μg/mL polybrene, and subjected to centrifugation at 1800 rpm for 45 minutes at 20°C. Following a 12-hour incubation post-spinfection, the medium was replaced, and cells were cultured for an additional 48 hours before further handling.

**Histology assays**

Heart specimens were fixed in 4% paraformaldehyde solution, dehydrated, and embedded in paraffin. Heart architecture was analyzed from transverse 4-mm-thick deparaffinized sections stained with hematoxylin&eosin staining (H&E) and sirius red staining in accordance with the manufacturer's protocol (Solarbio, Beijing, China). Briefly, the tissue was stained with hematoxylin for 3 min and rinsed under running water for 3 min and eosin for 2min. Drops of sirius red stain were added for 20 min. Photographs were taken using an Olympus FV1000 (Tokyo, Japan). Fibrotic area of heart was calculated by Image-Pro Plus 6.0 software (Media Cybernet ics, Inc, Rockville, MD, USA). Immunohistochemistry (IHC) for SENP1 and MEF2C was conducted using a microscope Olympus FV1000 (Tokyo, Japan). Quantification of IHC staining was carried using the Image J software.

**Triphenyltetrazolium-Chloride (TTC) Staining**

After the model was established, the cardiac tissue was extracted, cleaned in a PBS solution at 0-4°C, and then frozen at -20°C for 20 minutes. The tissue block was then cut to obtain coronal slices measuring 2 mm. These slices were immersed in a 2% TTC solution and water-bathed at 37°C in the dark for 30 minutes, with shaking at 5-minute intervals to ensure uniform staining. Following this, the stained tissue samples were fixed overnight with 4% paraformaldehyde and dried the next day for photography.

**Calculation of Myocardial Infarct Area**

The stained myocardial slices were placed on an EPSON scanner (V700) and scanned in 24-bit true color at a resolution of 2400 dpi. Traditional Method: Area statistical analysis was performed using Image-Pro Plus 6.0 to determine the left ventricular area (total myocardium, LV), the area at risk of ischemic (AAR), and the area of the infarct zone (IA). Quality Weighting Method: Each myocardial slice was weighed individually, and the percentage of each slice's weight relative to the total weight of all slices was calculated. Using the traditional method as a basis, the area of each region in each slice was multiplied by the corresponding weight percentage of that slice. The AAR/LV ratio was used to evaluate the consistency of the ligation position, indicating whether the ischemic risk zones experienced a uniform degree of ischemia, while the IA/AAR ratio was utilized to assess the extent of myocardial infarction.

**Measurement of cardiac function**

Four weeks after induced myocardial infarction, echocardiographic assessments were performed on mice using the Vevo2100 High-Resolution Imaging System from Visual Sonics, Toronto, ON, Canada. The system was equipped with a 10-MH2 phased-array transducer for M-mode recording. The mice were anesthetized with isoflurane (2.5% for induction and 0.5% for maintenance) and positioned on an electric heating pad within the Visual Sonics Vevo Integrated Rail System II to maintain a constant body temperature of 37°C. Two-dimensional targeted M-mode traces were obtained from the parasternal short-axis view at the mid-papillary muscle level and the parasternal long-axis view just below the papillary muscle. Left ventricular parameters, such as left ventricular end-diastolic volume (LVEDV), left ventricular end-systolic volume (LVESV), left ventricular internal dimension at end-diastole (LVIDd), and left ventricular internal dimension at systole (LVIDs), left ventricular end-diastolic diameter (LVEDD) and left ventricular end-systolic diameter (LVESD) were quantified from the M-mode recordings. The results were based on the average of measurements taken from three consecutive heartbeats. Ejection fraction (EF) was determined using the formula EF = (LVEDV - LVESV) / LVEDV × 100%, while fractional shortening (FS) was calculated as (LVIDd - LVIDs) / LVIDd × 100%. In addition, it includes the detection of heart rate. The serum concentrations of Cardiac Troponin level (cTnI) and Creatine Kinase MB (CK-MB) in the control mice and TKO mice or AAV9-Vec group and AAV9-SENP1 group were analyzed within 24 hours after blood collection.

**RNA-sequencing**

Heart tissues at marginal zone of infarcted area were taken for RNA-sequencing 14 days after the operation, and total RNA was extracted from three biological samples. The quality of RNA was evaluated with a 2100 Expert Bioanalyzer (Agilent) before being processed for library preparation and sequencing on the Illumina Hiseq2000 platform at Majorbio Biotech (Shanghai, China). Subsequently, the data was analyzed using the free online Majorbio I-Sanger Cloud Platform UENCING.

**RNA isolation and quantitative real-time PCR**

Total RNA was extracted using the RNeasy miniprep kit (Qiagen) following the manufacturer's protocol. Reverse transcription was conducted using the High-Capacity cDNA Synthesis Kit (Thermo-Fisher). Quantification of target gene expression was carried out using a SYBR Green PCR Master Mix (Applied Biosystems, PE, Foster City, CA, USA). Real-time PCR for relative mRNA expression was performed on the ABI PRISM 7700 Sequence Detection System (Applied Biosystems). Oligonucleotide primers were used to assess mRNA gene expression, with *Gapdh* serving as the internal control. The 2^-ΔΔCt^ formula was utilized to determine the fold change in gene expression, and the average ΔCt value from three biological replicates was recorded.

mouse *Gapdh*-F: TCAAGCTCATTTCCTGGTATGACA

mouse *Gapdh*-R: TAGGGCCTCTCTTGCTCAGT

mouse *Senp1*-F: CTCCCACCTGCTTCGCC

mouse *Senp1*-R: AGACACAGAATCCAAGAGGC

mouse *Senp2*-F: TAGCAGTGAGATACATTTCAAAGTG

mouse *Senp2*-R: GACCTTCAAAGAGAAACCTGAGTG

mouse *Senp3*-F: CTTATGGCAGCCTCATCCCTCT

mouse *Senp3*-R: TTGCCTGGCATCCGCTGATAAG

mouse *Senp5*-F: TGGAAGTCTGGTCCCACTCAGT

mouse *Senp5*-R: GGAAGTTACACTTTTGATGTCTGG

mouse *Senp6*-F: CAGTGATGACGGACTCCTTGCT

mouse *Senp6*-R: CTCTGAGTGAGTCCATAAGGAGG

mouse *Senp7*-F: GGAACTCCAAAAGGTTGTGTCAC

mouse *Senp7*-R: GTGGTCCTCATCCTTGCTTTCC

mouse *Myh6*-F: GCTGGAAGATGAGTGCTCAGAG

mouse *Myh6*-R: CCAGCCATCTCCTCTGTTAGGT

mouse *Myh7*-F: GCTGGAAGATGAGTGCTCAGAG

mouse *Myh7*-R: TCCAAACCAGCCATCTCCTCTG

mouse *Bnp*-F: TCCTAGCCAGTCTCCAGAGCAA

mouse *Bnp*-R: GGTCCTTCAAGAGCTGTCTCTG

mouse *Gata4*-F: GCCTCTATCACAAGATGAACGGC

mouse *Gata4*-R: TACAGGCTCACCCTCGGCATTA

mouse *Mef2c*-F: GTGGTTTCCGTAGCAACTCCTAC

mouse *Mef2c*-R: GGCAGTGTTGAAGCCAGACAGA

**Western blotting**

Procedures of Western blotting have been detailed in our previous study^1^. Briefly, protein homogenates were separated by SDS-PAGE and transferred to PVDF membranes, and then blocked with 5% skim milk for 1 h and incubated with anti-SENP1 (#25349-1-AP, Proteintech, 1:1000), anti-MEF2C (#5030, CST, 1:1000), anti-HA (#3724, CST, 1:1000), anti-FLAG (#A8592, Sigma-Aldrich, 1:5000), anti-c-PARP (#9541, CST, 1:1000), anti-c-Caspase3 (#9661, CST, 1:1000), anti-HSP90ab1 (#67450-1-Ig, proteintech, 1:1000), anti-SUMO2 (#PA5-110446, ThermoFisher Scientific, 1:1000), anti-αMHC (#MA5-35613, ThermoFisher Scientific, 1:1000), anti-βMHC (MYH7, #22280-1-AP, proteintech, 1:1000), anti-GATA4 (#sc-25310, Santa Cruz Biotechnology, 1:500), anti-GST (#ab184804, Abcam), anti-Ubiquitin (#3936, CST, 1:1000) and anti-β-actin (#AC006/AC026, Abclonal, 1:5000) at 4°C overnight. Subsequently, the membrane was incubated with goat anti-rabbit IgG H&L (HRP) (#A0545, ThermoFisher Scientific, 1:10000) and rabbit anti-mouse IgG H&L (HRP) (#A9044, ThermoFisher Scientific, 1:10000), and then visualized with the enhanced chemiluminescence reagents (#34580, ThermoFisher Scientific). At least three independent experiments were used for statistical analysis.

**Cycloheximide Chase Assay**

Cycloheximide chase assay was carried out according to a report^2^. Briefly, to equilibrate yeast cell suspensions, incubate for 5 minutes at 30 °C. Prepare a timer to count up from 0:00 to begin the cycloheximide chase. Start the cycloheximide chase by adding 250 µg/mL cycloheximide to the first cell suspension, vortex briefly, and transfer 950 µL of this mixture to a microcentrifuge tube with 50 µL of ice-cold 20 × stop mix. Vortex and place on ice, then return remaining suspension to 30 °C. Repeat the above steps for subsequent samples at regular intervals (e.g., every 30 seconds). For each time point, vortex the suspension, transfer 950 µL to labeled tubes with 50 µL stop mix, and return the remaining cells to the heat block. To prevent settling, vortex the suspensions every 5 minutes or maintain them in a continuously agitating water bath during the experiment.

**Co-immunoprecipitation (Co-IP)**

Cells were gathered and then lysed with NP-40 lysis buffer, which included complete protease inhibitors, while kept on ice for 30 minutes. The cell lysate was subsequently centrifuged at 12,000g for 20 minutes at a temperature of 4°C. To co-immunoprecipitate exogenously expressed proteins, the supernatant was incubated with anti-FLAG M2 Affinity Gel (Sigma-Aldrich, A2220) overnight at 4°C. The next day, the pellet was washed four times using NP-40 lysis buffer buffer before undergoing western blotting analysis.

**Sliver staining and mass spectrometry**

Protein of Co-IP samples was resolved in the gel, and then silver staining was performed according to the manufacturer’s instructions of Pierce™ Silver Stain Kit. Briefly, after fixing in 30% ethanol:10% acetic acid solution, washing in 10% ethanol and watery, sensitizing in sensitizer working solution, staining in stain working solution one by one, the gel containing protein was developed in developer working solution and stopped in 10% acetic acid solution until the protein bands appeared. Protein of specified samples were collected, and the corresponding bands were subjected to mass spectrometry assay as previously described after staining. The results of mass spectrometry analysis were presented in the supplementary table 2.

**GST-pulldown assays**

For protein purification, GST-SENP1 was constructed into pGEX-5X-3 vector, the plasmid and vector control were transformed into BL21 bacterial cells, and the fresh bacterial colony was induced by 0.2 mM IPTG at 16 °C overnight until the colony reached OD_600_ of 0.8-0.9. Pellets were lysed in GST buffer and centrifuged at 12,000g at 4 °C for 20 min. Supernatant was added with NaCl (500 mM) and glutathione agarose beads (200 μL) (Thermo Fisher, 16100), and incubated on suspension instrument at 4 °C overnight, and glutathione agarose beads were washed with GST buffer twice next day.

**Apoptosis assay**

The apoptosis assay was conducted using the Annexin V-FITC Apoptosis Detection Kit (Sigma-Aldrich, St. Louis, MO, USA) following the manufacturer's instructions. A total of 1×10^5^ cells were stained with 5 μL Annexin V-FITC and 1 μL of PI in the dark. The cells were then analyzed using the FACS Calibur instrument. Flow cytometry data was analyzed using CellQuest 3.0 software (BD Biosciences, New Jersey, USA). Cells that tested negative for both Annexin V and PI were classified as viable, those positive for Annexin V and negative for PI were deemed early apoptotic, and cells positive for both Annexin V and PI were considered late apoptotic.

**Immunofluorescence staining**

The cells were fixed in a 4% paraformaldehyde solution for 20 minutes at room temperature. Subsequently, the samples were treated with 0.5% Triton X-100 for 15 minutes and then blocked with 5% BSA for 30 minutes at room temperature. Following an overnight incubation at 4°C with SENP1 antibody (#PA1-41169, ThermoFisher Scientific, 1:100), MEF2C antibody (#sc-518152, Santa Cruz Biotechnology, 1:50) and cTnT (#sc-20025, Santa Cruz Biotechnology, 1:50), the samples were incubated with Goat anti-Mouse IgG (H+L) Highly Cross-Adsorbed Secondary Antibody, Alexa Fluor™ 555 (Invitrogen, A21424), Goat anti-Rabbit IgG (H+L) Highly Cross-Adsorbed Secondary Antibody, Alexa Fluor™ 488 (Invitrogen, A11034) (1:1000) or Goat Anti-Mouse IgG H&L (Alexa Fluor® 647) (abcam, ab150115) (1:1000) for 60 minutes in a dark environment at room temperature, followed by nucleus counterstaining with DAPI. Imaging was performed using the Olympus FV1000 IX81-SIM Confocal Microscope (Olympus, Tokyo, Japan), and further analysis was carried out using ImageJ.

**Fluorescence recovery after photobleaching (FRAP)**

H9c2 cells transfected with corresponding plasmids and the purified protein of interest were utilized in FRAP experiments conducted on an Olympus FV1000 IX81-SIM Confocal Microscope (Olympus, Tokyo, Japan). Photobleaching was carried out using the tornado mode with a 488 nm laser set at 45% power for GFP. The recovery of fluorescence was observed using the 488 nm laser in free-run mode at intervals of 1s to 4.2s. Additionally, fluorescence from an unbleached area within the same field was monitored as a control. The signal was quantified as a ratio relative to the fluorescence intensity prior to photobleaching.

**In vitro droplet assay**

To purify the protein of interest, plasmids harboring the fusion protein with GFP were transformed into BL21 cells. A fresh bacterial colony was inoculated into 200 mL LB media supplemented with kanamycin and incubated at 37℃ until reaching an OD600 of 0.8-0.9. IPTG was then added to a final concentration of 1 mM, and the culture was allowed to grow overnight at 16℃. The cell pellets from the 200 mL culture were resuspended in 10 mL of GST lysis buffer (50 mM Tris-HCl pH 7.5, 100 mM NaCl) containing 1 mM dithiothreitol, 0.2 mM phenylmethylsulfonyl fluoride, 1% Triton-X100, and complete protease inhibitor, followed by sonication (4 cycles of 30 seconds on, 30 seconds off). The lysate was clarified by centrifugation at 15,000 rpm for 20 minutes at 4℃, and NaCl was added to a final concentration of 500 mM. Subsequently, 500 μL of prewashed glutathione agarose (Thermo Fisher, 16100) was added to the lysate and the tubes were rotated at 4 ℃ overnight. The agarose was then washed twice with GST lysis buffer containing 500 mM NaCl and twice with GST lysis buffer. The protein was obtained by cleaving with PierceTM HRV 3C protease and assessed by Coomassie-stained gel.

For the droplet assay, protein was added to solutions at varying concentrations along with specified final salt and molecular crowder concentrations in Buffer A (50 mM Tris-HCl pH 7.5, 10% glycerol, 1 mM DTT). The protein solution was promptly loaded onto a custom chamber consisting of a glass slide attached to a coverslip using two parallel strips of double-sided tape. Subsequently, the slides were captured using an Andor confocal microscope equipped with a 100 × objective. Imaging was conducted using the Olympus FV1000 IX81-SIM Confocal Microscope (Olympus, Tokyo, Japan).

**Turbidity assay**

In this assay, the turbidity of the phase-separated solution was assessed by measuring absorbance at 600 nm using a NanoDrop spectrophotometer (Thermo Scientific). Protein samples were prepared by combining specified quantities of the target protein, NaCl, and buffer to attain the desired concentrations of each component. Absorbance at 600 nm was continuously monitored and recorded at room temperature using the NanoDrop. The recorded absorbance values were reported after subtracting the optical density of the buffer.

**Statistical analysis**

For comparisons between two groups, analyses were conducted using the unpaired Student's t-test (for normally distributed data) or the Mann–Whitney test (for non-normally distributed data). For comparisons involving multiple groups, one-way analysis of variance (ANOVA) was applied, followed by Tukey's post hoc test, or two-way ANOVA with Sidak's post hoc test. When comparing two conditions between groups, two-way ANOVA with the appropriate post-hoc correction was used. The correlation between gene expressions was determined by the Pearson correlation test, and survival analysis was performed using GraphPad Prism 5.0. All the experiments were biologically repeated in at least 3 independent samples, and technically repeated at least 3 times. Statistical significance was considered for *P* values less than 0.05.

References

1. Liu J, Xie Y, Guo J, et al. Targeting NSD2-mediated SRC-3 liquid-liquid phase separation sensitizes bortezomib treatment in multiple myeloma. *Nat Commun*. Feb 15 2021;12(1):1022. doi:10.1038/s41467-021-21386-y

2. Buchanan BW, Lloyd ME, Engle SM, Rubenstein EM. Cycloheximide Chase Analysis of Protein Degradation in Saccharomyces cerevisiae. *J Vis Exp*. Apr 18 2016;(110)doi:10.3791/53975
