# Supplementary material for: Downregulation of SENP1 impairs nuclear condensation of MEF2C and deteriorates ischemic cardiomyopathy
Source: Clin Transl Med. 2025 May 7;15(5):e70318. doi: 10.1002/ctm2.70318 (PMC12059206; doi:10.1002/ctm2.70318)
Supplement: Supplementary file 2 — Supporting Information [file CTM2-15-e70318-s004.docx]

**Supplementary figures**


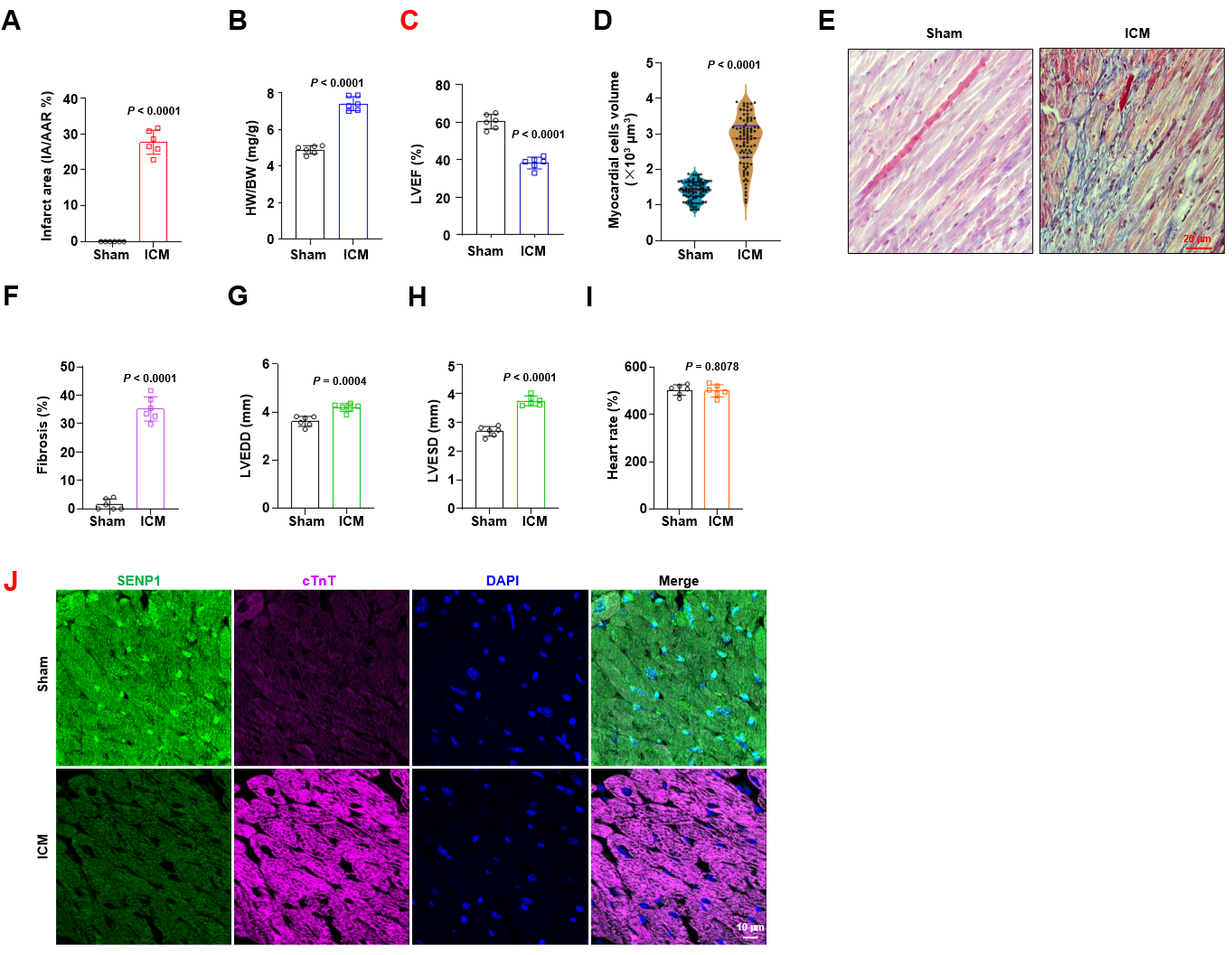


**Figure S 1. Parameters of impaired cardiac function in ICM mouse model.**

**(A)** Percentage of infarct area (IA/AAR), **(B)** ratio of heart-weight/body-weight (HW/BW), **(C)** left ventricular ejection fraction (LVEF) and **(D)** myocardial cells volume, as compared with the mock controls of sham mice and ICM mice (n = 6/group). **(E)** Representative images of sirius red staining of heart tissue from ICM or sham mice, scale bar, 20 μm, and **(F)** quantification for the percentage of fibrosis (n = 6/group). **(G)** Left ventricular end-diastolic diameter (LVEDD), **(H)** left ventricular end-systolic diameter (LVESD), and **(I)** heart rate percentage of ICM or sham mice (n = 6/group). **(J)** Immunofluorescence staining for SENP1 and cTnT protein levels in serial sections of heart tissues from ICM model and sham group. Scale bar, 10μm. Two-sided *P* values were determined by Student’s t-test. Data indicates mean ± SEM.


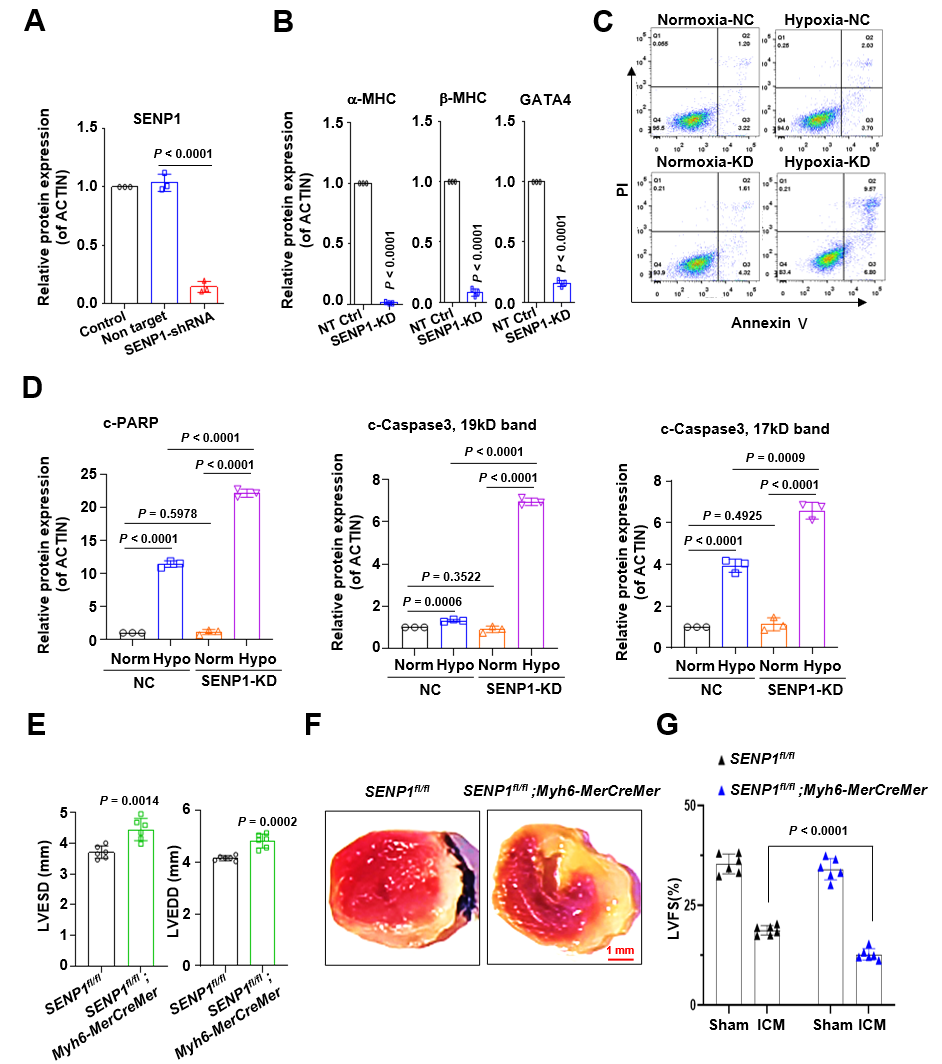


**Figure S 2. Knockdown of SENP1 exacerbates myocardial injury.**

**(A)** Quantification of Fig 2A (n = 3). **(B)** Quantification of Fig 2B (n = 3). **(C)** Flow cytometry analysis of the apoptotic H9c2 cells after SENP1 knockdown under hypoxic condition (n = 3 each group). **(D)** Quantification of Fig 2D (n = 3). **(E)** Left ventricular end-systolic diameter (LVESD) and left ventricular end-diastolic diameter (LVEDD) of *Senp1*^fl/fl^ and *Senp1*^fl/fl^; *Myh6-MerCreMer* (*Senp1*-TKO) mice (n = 6/group). **(F)** TTC staining of heart tissues from *Senp1*^fl/fl^ and *Senp1*^fl/fl^; *Myh6-MerCreMer* (*Senp1*-TKO) mice. **(G)** Percentage of left ventricular fractional shortening (LVFS) of control mice and TKO mice under ICM conditions (n = 6/group). Two-sided *P* values were determined by Student’s t-test. Data indicates mean ± SEM.


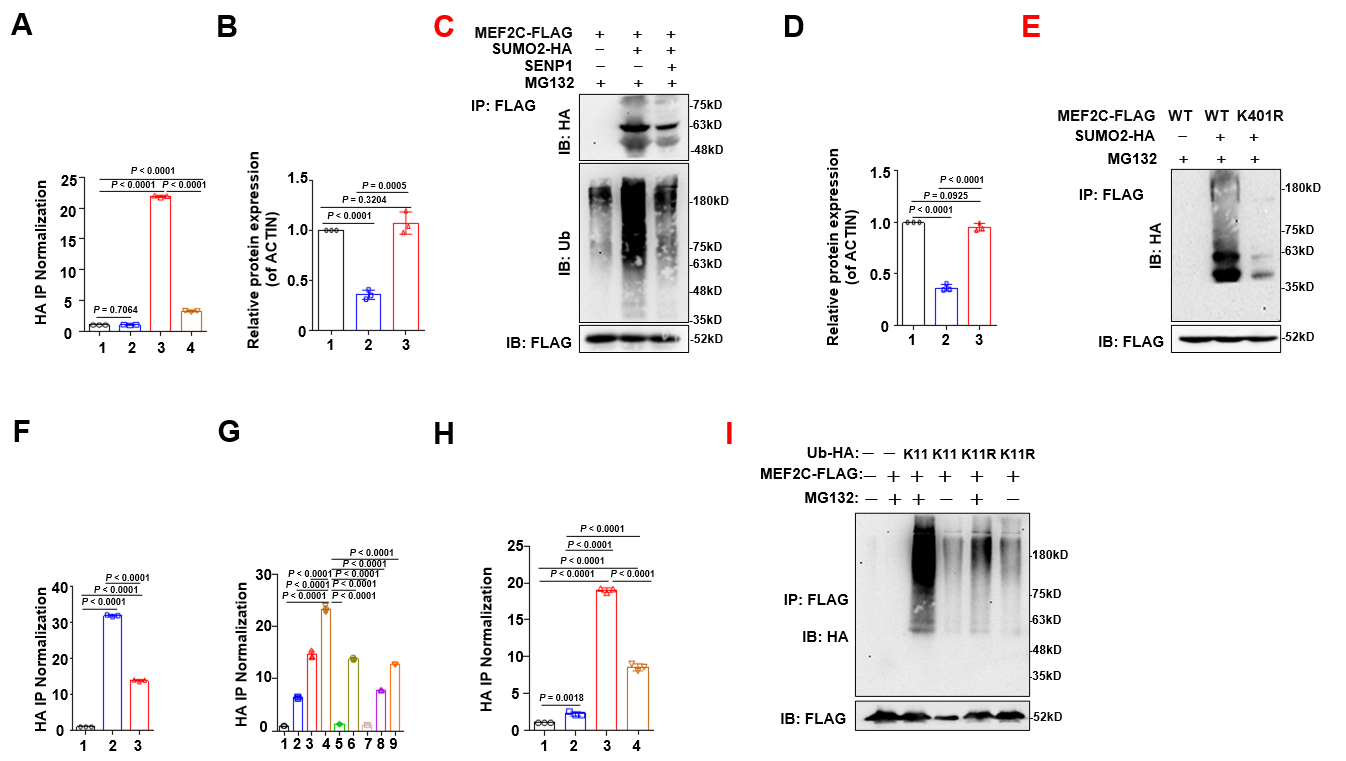


**Figure S 3. Quantitative western blot analysis.**

**(A)** Quantification of Fig 4F, 4I **(B)**, 4J **(D)**, 4K **(F)**, 4L **(G)**, 4M **(H)** (n = 3). **(C)** Co-IP assay showing SUMOylation and ubiquitination levels of MEF2C protein in HEK293T cells after MEF2C and SUMO2 or SENP1 overexpression in the presence of MG132. **(E)** Co-IP assay showing SUMOylation levels of FLAG-tagged MEF2C^Wt^ or MEF2C^K401R^ co-expressing with SUMO2 in HEK293T cells in the presence of MG132. **(I)** Co-IP assay validating the K11-ubiquitin conjugated ubiquitination of MEF2C protein. K11R, ubiquitin contains all lysine residues except the lysine residue at position 11 was mutated. Two-sided *P* values were determined by Student’s t-test. Data indicates mean ± SEM.


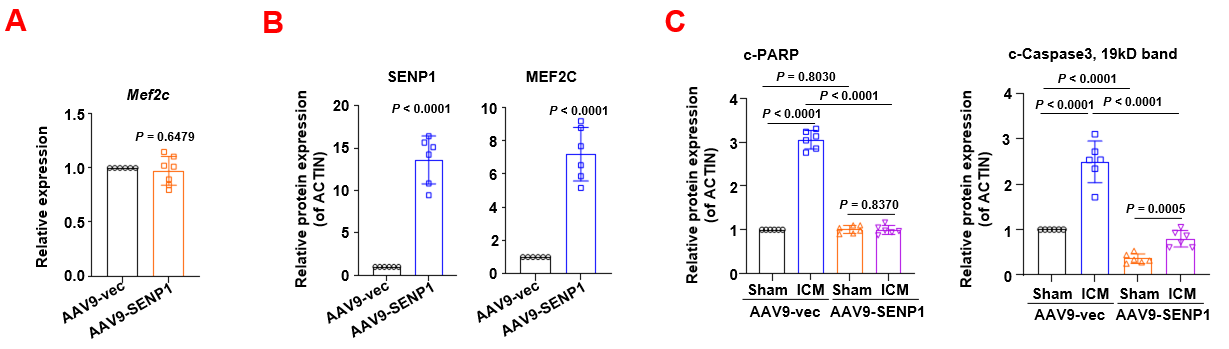


**Figure S 4. Overexpression of SENP1 improves ICM in vivo. (A)** qRT-PCR analysis for *Mef2c* in the AAV9-SENP1 and AAV9-vector mice (n = 6). **(B)** Quantification of Fig 7C (n = 6). **(C)** Quantification of Fig 7G (n = 6). Two-sided *P* values were determined by Student’s t-test. Data indicates mean ± SEM.
