# Supplementary material for: Downregulation of SENP1 impairs nuclear condensation of MEF2C and deteriorates ischemic cardiomyopathy
Source: Clin Transl Med. 2025 May 7;15(5):e70318. doi: 10.1002/ctm2.70318 (PMC12059206; doi:10.1002/ctm2.70318)
Supplement: Supplementary file 3 — Supporting Information [file CTM2-15-e70318-s001.docx]

**Table 1. Clinical characteristics**

| Pt No./  Gender | Age  (yr) | Disease | HR  (beats/min) | PR  Interval  (ms) | QRS Interval  (ms) | QT/QTc  Interval  (ms) |
| --- | --- | --- | --- | --- | --- | --- |
| 1/F | 45 | Ischemic cardiomyopathy；  Ventricular aneurysm | 65 | 112 | 130 | 445/457 |
| 2/M | 60 | Ischemic cardiomyopathy；  Ventricular aneurysm | 80 | 81 | 91 | 370/380 |
| 3/F | 51 | Ischemic cardiomyopathy；  Ventricular aneurysm | 77 | 69 | 102 | 420/436 |
| 4/M | 49 | Ischemic cardiomyopathy；  Ventricular aneurysm | 78 | 123 | 128 | 450/461 |
| 5/F | 61 | Ischemic cardiomyopathy；  Ventricular aneurysm | 67 | 74 | 111 | 436/451 |
| 6/M | 44 | Ischemic cardiomyopathy；  Ventricular aneurysm | 86 | 92 | 96 | 390/413 |

F= female; HR = heart rate; M=male; Pt=patient; QTc=heart-rate corrected QT interval.
